# Tahyna Virus and Human Infection, China

**DOI:** 10.3201/eid1502.080722

**Published:** 2009-02

**Authors:** Zhi Lu, Xin-Jun Lu, Shi-Hong Fu, Song Zhang, Zhao-Xia Li, Xin-Hua Yao, Yu-Ping Feng, Amy J. Lambert, Da-Xin Ni, Feng-Tian Wang, Su-Xiang Tong, Roger S. Nasci, Yun Feng, Qiang Dong, You-Gang Zhai, Xiao-Yan Gao, Huan-Yu Wang, Qing Tang, Guo-Dong Liang

**Affiliations:** Chinese Center for Disease Control and Prevention, Beijing, People’s Republic of China (Z. Lu, X.-J. Lu, S.-H. Fu, D.-X. Ni, F.T. Wang, Y. Feng, Y.-G. Zhai, X.-Y. Gao, H.-Y. Wang, Q. Tang, G.-D. Liang); Xinjiang Center for Disease Control and Prevention, Xinjiang, People’s Republic of China (S. Zhang, S.-X. Tong, Q. Dong); Kashi Center for Disease Control and Prevention, Xinjiang (Z.-X. Li); Jiashi County Center for Disease Control and Prevention, Xinjiang (X.-H. Yao); Maigaiti County Center for Disease Control and Prevention, Xinjiang (Y.-P. Feng); Centers for Disease Control and Prevention, Fort Collins, Colorado, USA (A.J. Lambert, R.S. Nasci)

**Keywords:** Tahyna virus, California group viruses, Bunyaviridae, China, dispatch

## Abstract

In 2006, Tahyna virus was isolated from *Culex* spp. mosquitoes collected in Xinjiang, People’s Republic of China. In 2007, to determine whether this virus was infecting humans, we tested serum from febrile patients. We found immunoglobulin (Ig) M and IgG against the virus, which suggests human infection in this region.

Tahyna virus (TAHV) has been reported to cause human illness throughout much of Europe and Asia, including countries adjacent to the border of northwestern China (*1*–*4*). TAHV (family *Bunyaviridae,* genus *Orthobunyavirus*, California serogroup) was first isolated in the former Czechoslovakia in 1958 from a pool of *Aedes caspius* mosquitoes (*5*). TAHV is widely distributed in central Europe, as shown by the following: numerous virus isolates have been obtained from mosquitoes, TAHV-specific antibodies have been detected in several nonhuman mammals and several avian species, and TAHV antibodies are highly prevalent in humans in some localities (*6*). Human illness from infection with TAHV has been reported as manifesting undifferentiated fever and influenza-like symptoms; the infection may also cause pneumonia and pleurisy, acute arthritis, pharyngitis, and, occasionally, central nervous system involvement (*6*,*7*).

The presence of TAHV in China was postulated after antibodies were detected in serum specimens collected from healthy adults in Xinjiang Province in 1985 (*8*), although a subsequent study of the cause of encephalitis cases across China during 1988–1990 failed to detect seroconversion to any California group viruses (*9*). Here we report isolation of TAHV in China, along with evidence of TAHV infections in humans. Human infection can be inferred from positive serologic results of samples taken from persons who visited an outpatient clinic in the same region from which the TAHV isolate was derived.

## The Study

**Figure 1:**
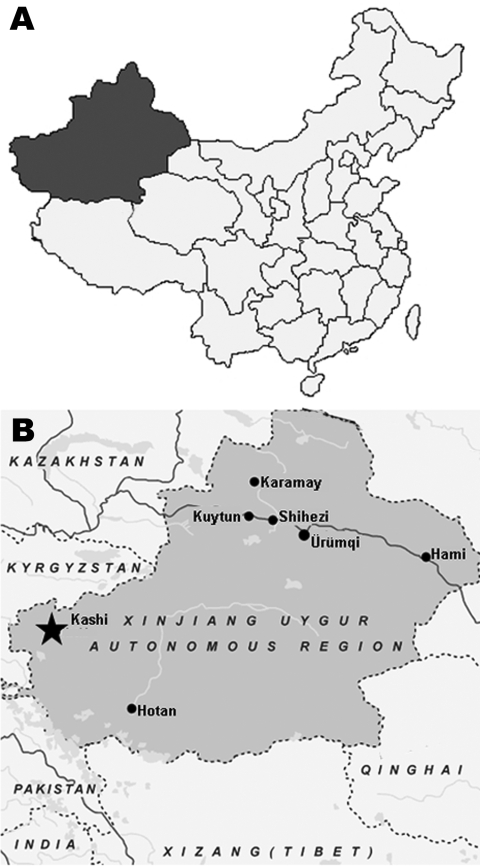
A) Map of China showing location of Xinjiang Uygur Autonomous Region. B) Map of Xinjiang Uygur Autonomous Region showing Kashi region (star), where Tahyna virus XJ0625 was isolated from a pool of *Culex* spp. mosquitoes.

Mosquitoes were collected in several counties in the Kashi area, Xinjiang Uygur Autonomous Region, China (Figure 1, panel A), from July through August 2006. Mosquitoes were collected in light traps, identified, and separated by genus/species/collection site into pools of up to 100 specimens. Species collected included *Culex annulirostris, Cx. quinquefasciatus,* and *Cx. pipiens.* Unfortunately, several specimens could not be identified below genus. A total of 9,865 mosquitoes were tested in 100 pools that contained both unfed and blood-fed specimens.

Specimens were triturated in minimal essential medium, clarified by centrifugation, and added to confluent sheets of baby hamster kidney (BHK)–21 and Vero cells in 6-well plates, which were incubated at 37°C (*10*). A single pool containing 100 specimens of unidentified *Culex* spp. mosquitoes collected from Tierimu town, Jiashi county, Kashi (39°52′87′′N, 76°71′51′′E) (Figure 1, panel B), yielded a virus isolate designated XJ0625. Because the pool contained unfed and blood-fed mosquitoes, the virus isolate may have come from either an infected mosquito or from infected animal blood in the mosquito’s gut. At 24 h after the mixture was added to the 6-well plates, this isolate produced giant cells and syncytia in both BHK and Vero cells. The cell monolayer was destroyed rapidly, within 24 h of the first cytopathic effects. After intracranial inoculation with XJ0625, suckling mice showed signs of tremor and stiff neck at 24 h and died within 48 h.

RNA was extracted from an XJ0625 RNA lysate and subjected to reverse transcription–PCR (RT-PCR) amplification by using genus primer sets designed for the detection of flavivirus, alphavirus, and bunyavirus RNA (*11*–*13*). The sequence amplified by the bunyavirus genus primer had a high homology with TAHV. Subsequently, microplate plaque-reduction neutralization tests (*14*) were performed with BHK-21 cells and immune ascites fluid with immunity to prototype TAHV (Bardos 92; provided by the Centers for Disease Control and Prevention (CDC), Fort Collins, CO, USA) to validate the molecular identification. XJ0625-associated cytopathic effects were completely inhibited at ascites fluid dilutions up to 1:3,200.

The nucleotide sequence of the small (S) (EU622820) and medium (M) (EU622819) segments of XJ0625 were sequenced by using the primers SF (5′-AGTAGTGTACCCCACTTGAAT AC-3′), SR (5′-CAAATGGATTTGATCCTGATGC-3′), M1F (5′-CACAAGTTCCAAGA TGATGTT-3′), M 1R (5′-CTGTGCCTTCTGCTTGGACTA-3′), M2F (5′-GTCCAAGC AGAAGGCACAGAT-3′), M2R (5′-GTGGTCACTGTACATTCTCCTGAA-3′), M3F (5′-CACACTTCTGTTTAGCAGATACC-3′), M 3R (5′-CTCTAGTCTATAGCTTGCTG GTGTT-3′), M4F (5′-GCACCAATCTGAACGCAATAACAC-3′), and M4R (5′-AGTAG TGTGCTACCAAGTATA-3′). By using Clustal X version 1.8 (www.clustal.org), sequences were aligned with those of viruses belonging to the California virus group. The phylogenic status of XJ0625 isolate was assessed by using MEGA version 3.1 software (www.megasoftware.net), and phylogenetic trees were constructed by using the neighbor-joining algorithm with 1,000 bootstrap replicates.

**Figure 2:**
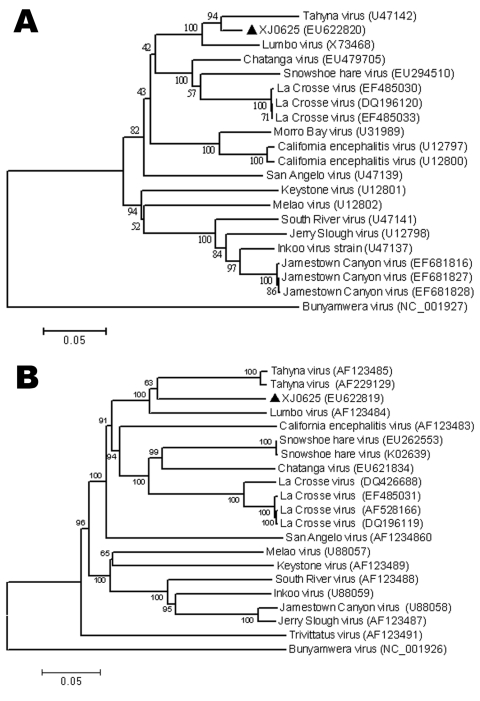
Phylogenetic analysis of Tahyna virus (TAHV) XJ0625 from China based on the complete nucleotide sequence of the small segment (A) and the medium segment (B). Distances and groupings were determined by the p-distance algorithm and neighbor-joining method with MEGA version 3.1 software (www.megasoftware.net). Bootstrap values are indicated and correspond to 1,000 replications. The tree was rooted by using Bunyamwera virus as the outgroup virus. Scale bars indicate a genetic distance of 0.05-nt substitutions per position.

Phylogenetic analyses of the nucleotide sequences of the S (Figure 2, panel A) and M (Figure 2, panel B) segments generated highly comparable topologies, which indicates that XJ0625 has a high level of sequence homology with TAHV. To provide independent confirmation, we sent the XJ0625 viral RNA to CDC, Fort Collins, Colorado, USA, for further characterization. RNA was subjected to RT-PCR amplification by using multiple primer sets designed for the detection of orthobunyavirus S segment RNA (*11*). Nucleotide sequencing of amplified DNA fragments that, in combination, span the entirety of the TAHV S segment was used to positively identify XJ0625 viral RNA as TAHV RNA (data not shown) (*15*).

To determine whether persons in the region were becoming infected with TAHV, we collected serum samples from 323 persons who visited an outpatient clinic in Jiashi County and its adjacent counties in Kashi from August 18 through September 20, 2007. Specimens were collected within 1–3 days of onset of clinical signs and symptoms, which consisted of fever (37°C–39°C) in all patients and headache with no other specific symptoms in a few. The serum specimens were screened by indirect immunofluorescence assay by using XJ0625-infected BHK-21 cells. Infected and uninfected cell suspensions were applied to Teflon-coated, 10-well slides, air dried, and fixed. The serum specimens were applied to the spot slides at dilutions of 1:40 for IgM detection and 1:80 for IgG detection. After incubation and washing, the spot slides were treated with fluorescein-conjugated antihuman IgM or IgG, dried, and examined by fluorescent microscope. Of the 323 samples, 42 (13.0%) were IgG positive and 17 (5.3%) of 323 were positive for both IgM and IgG against XJ0625 (Table).

**Table:** Results of serologic testing of human serum samples for the presence of antibody against TAHV XJ0625, 2007*

Case no.	Sex/age, y	Dates of serum sample collections	Days between serum sample collections	IgM (IFA)	IgG (IFA)	Neutralizing antibody titer
1	M/20	Sep 5	103	+	+	80
		Dec 17		NT	NT	40
2	M/72	Sep 5	103	+	+	80
		Dec 17		NT	NT	80
3	M/7	Sep 5	104	+	+	40
		Dec 18		NT	NT	10
4	M/25	Sep 6	103	+	+	80
		Dec 18		NT	NT	80
5	F/27	Sep 6	103	+	+	40
		Dec 18		NT	NT	40
6	M/35	Aug 18	122	+	+	40
		Dec 18		NT	NT	40
7	M/7	Aug 24	116	+	+	40
		Dec 18		NT	NT	80
8	F/43	Aug 27	113	+	+	40
		Dec 18		NT	NT	40
9	F/60	Aug 31	111	+	+	80
		Dec 20		NT	NT	80
10	F/42	Sep 16	95	+	+	80
		Dec 20		NT	NT	80

*TAHV, Tahyna virus; Ig, immunoglobulin; IFA, immunofluorescence assay; NT, not tested.

To determine whether the initial illness of the patients was associated with TAHV infection, in December 2007 (95–127 days after collection of the first sample) we obtained a second serum sample from 10 of the IgM-positive patients. These paired serum specimens were tested by serum dilution neutralization test with XJ0625 virus on BHK-21 cells. Serial 2-fold dilutions of serum were added to equal volumes of culture medium containing XJ0625 virus (50% tissue culture infective dose on a 96-well microtiter plate and incubated at 37°C for 1 h; 100 μL of each virus plus diluted serum was added to 4 wells of a 96-well tissue culture plate containing confluent monolayer of BHK-21 cells. After the cultures were incubated for 48 h at 37°C, we calculated the antibody titer as the highest dilution at which cytopathic effects were completely inhibited in the well. Most of the samples contained neutralizing antibody at a titer of 40 or 80 (Table), though no samples produced a 4-fold titer change that would suggest that TAHV infection caused the initial illness.

## Conclusions

We isolated TAHV from mosquitoes in China and detected IgG consistent with TAHV antibodies in 13.0% of the samples, which suggests that human infection with TAHV is common in the area. We recognize that further testing is necessary to determine whether this serologic response was due to TAHV or to another California serogroup orthobunyavirus.

These results indicate that in northwestern China, TAHV should be investigated as a possible etiologic agent in cases of febrile illness with pulmonary involvement and in cases with central nervous system involvement not attributable to other causes. Research should be conducted to identify the primary mosquito vectors and vertebrate amplifier hosts in this region. Additionally, TAHV should be investigated as a cause of illness in other areas of China.
